# Supplementary material for: Proximity labeling at H3K9me3 reveals VRK-1 regulate global chromatin distribution in C. elegans
Source: bioRxiv. 2026 Jul 10:2026.07.07.737140. Preprint. [Version 1] doi: 10.64898/2026.07.07.737140 (PMC13370418; doi:10.64898/2026.07.07.737140)
Supplement: 1 [file NIHPP2026.07.07.737140v1-supplement-1.pdf]

**Supplementary information**

**Strains used in this study**

**Supplementary table 1 - *C. elegans* strains and genotypes used in this study**

| Strain | Genotype | Source |
|---|---|---|
| PMW738 | *ubsSi35[dpy-30p::Cbx1_2::mScarlett; unc-119+ @ttTi5605] II; unc-119 (ed3) III.* | This study |
| PMW1259 | *his-72 (uge30[gfp::his-72]) III.* | Gift from Florian Steiner |
| PMW1339 | *ubsSi35[dpy-30p::Cbx1_2::mScarlett; unc-119+ @ttTi5605] II.*<br>*unc-119 (ed3) III; his-72 (uge30[gfp::his-72]) III.* | This study |
| PMW1043 | *ubsSi36[dpy-30p::Cbx1_2::BASU; unc-119+ @ ttTi5605]; pod-2 (syb1772[pod-2::His10]) II.; mccc-1 (syb1666[mccc-1::His10]) IV.; pyc-1 (syb1680[pyc-1::His10]) V.; pcca-1 (syb1626[pcca-1::His10]) X* | This study |
| PMW1044 | *ubsSi38[dpy-30p:: (ubs36 NLS)::BASU;Cb unc-119 @ ttTi5605]; pod-2 (syb1772[pod-2::His10]) II.; mccc-1 (syb1666[mccc-1::His10]) IV.; pyc-1 (syb1680[pyc-1::His10]) V.; pcca-1 (syb1626[pcca-1::His10]) X.* | This study |
| PMW743 | *ubsSi35[dpy-30p::Cbx-1_2::mScarlett; Cb unc-119+ @ttTi5605] II*<br>*gwIs4[baf-1::GFP-LacI;myo-3::RFP] X*<br>*unc-119 (?) III* | This study |
| PMW1039 | *vrk-1 (bq28[vrk-1::wrm>Scar>let]) II* | This study |
| PMW1341 | *vrk-1 (bq28[vrk-1::w>Scar>let]) II; his-72 (uge30[gfp::his-72]) III.* | This study |
| PMW1223 | *vrk-1 (bq28[vrk-1::degron::w>Scar>let]) II.; wrdSi23[eft-3p::TIR1::F2A::BFP::AID*::NLS::tbb-2 3'UTR] I:-5.32* | This study |
| PMW1289 | *his-72 (uge30[gfp::his-72]) III.; vrk-1 (bq28[vrk-1::degron::w>Scar>let]) II.; wrdSi23[eft-3p::TIR1::F2A::BFP::AID*::NLS::tbb-2 3'UTR] I:-5.32* | This study |
| PMW1447 | *his-72 (uge30[gfp::his-72]) III.*<br>*vrk-1 (ok1181)/mIn1[mIs14 dpy-10 (e128)] II;* | This study |

| | | |
|---|---|---|
| | *bqSi156[p1028 (unc-119 (+) vrk-1p::vrk-1 (K169E)::mCherry)] IV* | |
| PMW1397 | *vrk-1 (ok1181)/mIn1[mIs14 dpy-10 (e128)] II; bqSi156[p1028 (unc-119 (+) vrk-1p::vrk-1 (K169E)::mCherry)] IV* | *(Dobrzynska and Askjaer 2016)* |
| PMW1376 | *cshIs128 [rpl-28p::TIR1::T2A::mCherry::HIS-11)] II; lin-12 (ljf33[lin-12::mNeonGreen[C1]::loxP::3xFLAG::AID*]) III; lag-2 (bmd204[lag-2::mTurquoise2::lox511i::2xHA]) V* | CGC |

**Primers used in this study**

| **Name** | **Sequence (5'-3')** | **Usage** | **Source** |
|---|---|---|---|
| rPM1086 | ATGCCTAAAGATCCAGCCAA ACCTCCG | Degron primer | This study |
| rPM2050 | CTTCACGAACGCCGCCGC | Degron primer | This study |
| rPM1888 | CTACACCGTCGTCGAGCAAT ACGAGCGTTCCGAGGGACG TCACTCCACCGGAGGAATGG ACGAGCTCTACAATATGCCTA AAGATCCAGCCAAACCTCCG | Degron forward primer with 5' VRK-1::wrmScarlet homology | This study |
| rPM1889 | GGAAAATAGACACGTTAGAG GAATCAATAGACGAAATTATG GGATGCAATGTGACTCGATT GAAGATTCCAATGGTTTAACC CTTCACGAACGCCGCCGC | Degron reverse primer with 3' VRK-1::wrmScarlet homology | This study |
| rPM1890 | CCAGTGCGAAGTATAAAGAA CG | VRK-1 WT, VRK-1::wrmScarlet, VRK-1::wrmScarlet:: degron genotyping primer | This study |
| rPM1891 | CCTGTTGAGAGCTCGAAAC | VRK-1 WT, VRK-1::wrmScarlet, VRK-1::wrmScarlet:: degron genotyping primer | This study |
| rPM120 | CCACAGAGTAGTCCAGAAAA | *set-25* genotyping | (Towbin et al. 2012) |
| rPM121 | TTGGGGGAAATAGATTTTGG | *set-25* genotyping | (Towbin et al. 2012) |
| rPM122 | GAGAAATTGTCATTCGAGAG | *set-25* genotyping | (Towbin et al. 2012) |
| rPM1736 | CCAGTGCACTTTTCGAGTGG | CBX1_2x::NLS::BAS | This study |

| | | | |
|---|---|---|---|
| | | U, NLS::BASU genotyping | |
| rPM1737 | CGTGGTAGATGAGGTGTTGTCC | CBX1_2x::NLS::BASU, NLS::BASU genotyping | This study |
| rPM1803 | CAGGAGCAATCAAGAACGTCAAC | *pcca-1::His*$^{10}$ genotyping | (Artan et al. 2022) |
| rPM1807 | GGCTTGAAATTTGAGAAACTGCG | *pcca-1::His*$^{10}$ genotyping | (Artan et al. 2022) |
| rPM1802 | CATTCTGCCGTCGCACCAATG | *mccc-1::His*$^{10}$ genotyping | (Artan et al. 2022) |
| rPM1806 | CTGAGCTGCACAACCGTACTC | *mccc-1::His*$^{10}$ genotyping | (Artan et al. 2022) |
| rPM1805 | GGACGAATTCAGAAGCGGTG | *pyc-1::His*$^{10}$ genotyping | (Artan et al. 2022) |
| rPM1809 | GGCAAAGAGAAGGTGGTGGC | *pyc-1::His*$^{10}$ genotyping | (Artan et al. 2022) |
| rPM1804 | GTGCAACCGAACAGCTCAATG | *pod-1::His*$^{10}$ genotyping | (Artan et al. 2022) |
| rPM1808 | GGCGGGGAGCTAAGAATTTTC | *pod-1::His*$^{10}$ genotyping | (Artan et al. 2022) |
| oBT471 | ACGTCTACCCATGGATCGAG | *eft-3p::TIR1* genotyping | Gift from Benjamin Towbin |
| oBT472 | TGTAGGTGTCTGGGAAGTGG | *eft-3p::TIR1* genotyping | Gift from Benjamin Towbin |
| rPM1727 | GACTACAAGGACGACGACGACAAGGACGAaGAGTCCGGACCCAAAAAGAAACGCAAGGTTTCCGTCTCCGGAGGAGACGGAAAGCTCTCCGAGTCCGAGA | Repair template for NLS insert on *dpy-30p::BASU* | This study |
| rPM485 | ATTTTGTGGTATAAAATAGCCGAGTTAGGAAACAAATTTTTCTTTCAGGTTTCTCAGTAGTGACCATGTGCGTGGATCTTGCGTCCACACATCTCAAGGCGTACTT | *unc-58* repair template | (Arribere et al. 2014) |
| rPM484 | CACTTGAACTTCAATACGGCAAGATGAGAATGACTGGAAACCGTACCGCATGCGGTGCCTATGGTAGCGGAGCTTCACAT | *dpy-10* repair guide | (Arribere et al. 2014) |

| | | | |
|---|---|---|---|
| | GGCTTCAGACCAACAGCCTAT | | |
| B979 | TTGTGATGATTCGAGCTGCTCGT | *vrk-1::wrmScarlet* tagging | This study |
| B980 | AACACGAGCAGCTCGAATCATCA | *vrk-1::wrmScarlet* tagging | This study |
| B981 | TGGCTTGCTTCTCGTCAAACAAAGCCAACATTTGATGATTCGAGCTGCTCGTCAGAAGTG | *vrk-1::wrmScarlet* tagging | This study |
| B982 | CGCCACTTCTGACGAGCAGCTCGAATCATCAAATGTTGGCTTTGTTTGACGAGAAGCAAG | *vrk-1::wrmScarlet* tagging | This study |
| B983 | GGTTAAACCATTGGAATCTTCAATCGAGTCACATTGCATCCCATAATTTCGTCTATTGAT | *vrk-1::wrmScarlet* tagging | This study |
| B984 | TACATCAATAGACGAAATTATGGGATGCAATGTGACTCGATTGAAGATTCCAATGGTTTA | *vrk-1::wrmScarlet* tagging | This study |
| rPM2292 | TGTAATTATTTTGTGTTTCAGAAACCATGgcagccgctGTTAAGCATCGTGAaTTtGTCGGtGAaCCcATGGGCGACAAAGAAGTCACATGCATCGCCGGGATCGGGCCAACATATGGCA | *baf-1* 3A repair template | This study |
| rPM2293 | CGATTATTTTAGAGGAGAGGC | *baf-1* 5’ region forward primer | This study |
| rPM2294 | GGAACAAATGGTGTTTTTCG | *baf-1* 5’ region reverse primer<br><br>*(WT amplicon is cleaved by AccI, while the 3A amplicon is not) | This study |

**CRISPR guides used in this study**

| Name | Sequence (5'-3') | Usage | Source |
|---|---|---|---|
| cPM077 | GGAATGGACGAGCTCTACAA | CRISPR guide targeting *vrk-1::wrmScarlet* | This study |
| cPM055 | GGACGACGACGACAAGGACG | CRISPR guide for NLS insert on *dpy-30p::BASU* | This study |
| cPM122 | GAGTTCGTCGGAGAGCCAAT | *baf-1* N-terminus guide | This study |
| cPM004 | GTCCACGCACATGGTCACTA | *unc-58* co-CRISPR guide | (Arribere et al. 2014) |
| cPM001 | GCTACCATAGGCACCACGAG | *dpy-10 co-CRISPR* guide | (Arribere et al. 2014) |

**Plasmids used in this study**

| Name | Genotype | Backbone | Source |
|---|---|---|---|
| pPM495 | *dpy-30p::CBX1_2xChromo::mScarlet::tbb-2 3' UTR* | pCFJ151 | This study |
| pPM497 | *dpy-30p::CBX1_2xChromo::BASU::tbb-2 3' UTR* | pCFJ151 | This study |
| pPM501 | *dpy-30p::BASU::tbb-2 3' UTR* | pCFJ151 | This study |

## Supplementary figures legends

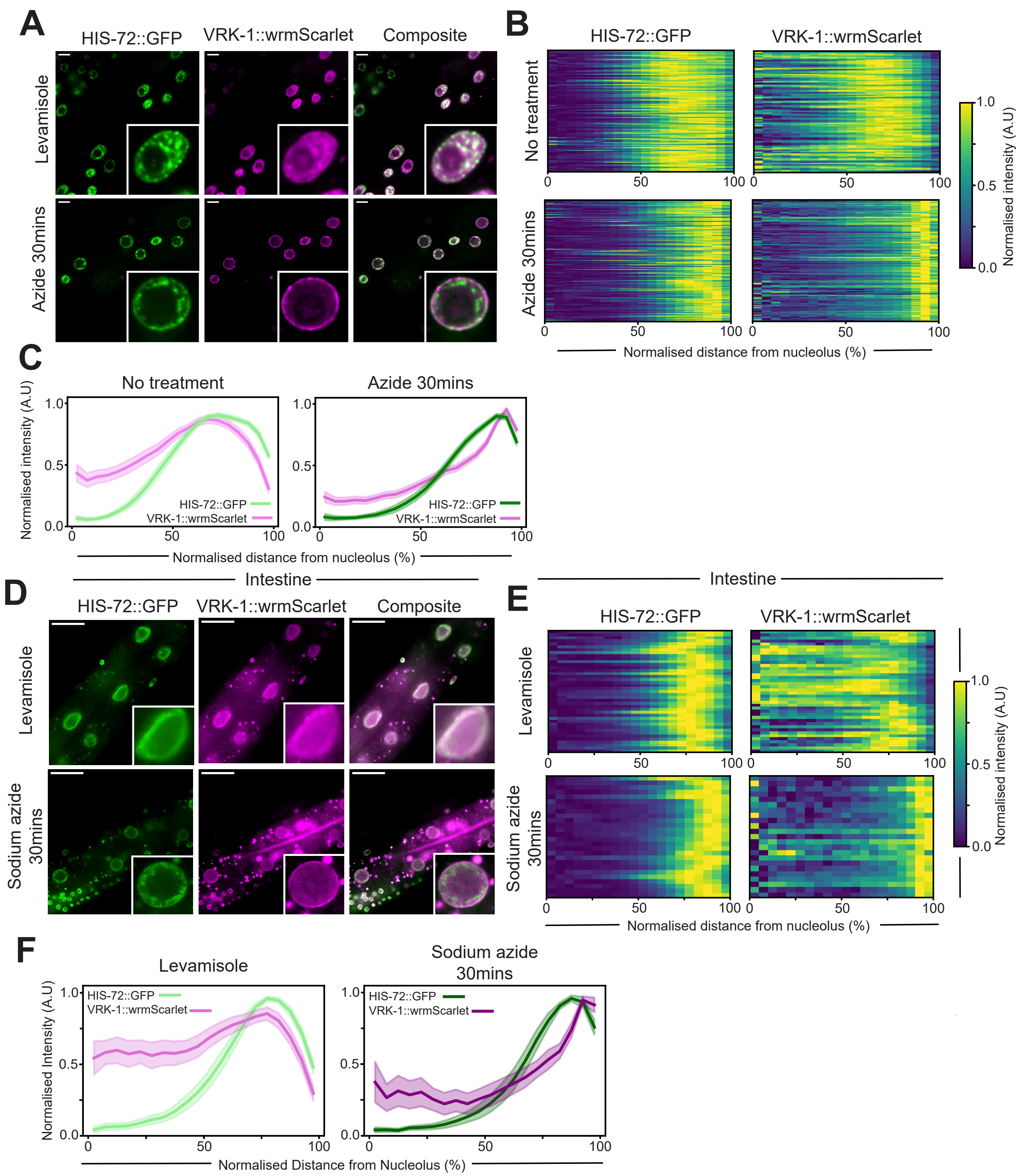

A
HIS-72::GFP
VRK-1::wrmScarlet
Composite
Levamisole
Azide 30mins
B
HIS-72::GFP
VRK-1::wrmScarlet
No treatment
Azide 30mins
Normalised distance from nucleolus (%)
Normalised intensity (A.U)
C
No treatment
Azide 30mins
Normalised intensity (A.U)
HIS-72::GFP
VRK-1::wrmScarlet
Normalised distance from nucleolus (%)
Intestine
D
HIS-72::GFP
VRK-1::wrmScarlet
Composite
Levamisole
Sodium azide 30mins
E
HIS-72::GFP
VRK-1::wrmScarlet
Levamisole
Sodium azide 30mins
Normalised intensity (A.U)
Normalised distance from nucleolus (%)
F
Levamisole
Sodium azide 30mins
Normalised Intensity (A.U)
HIS-72::GFP
VRK-1::wrmScarlet
Normalised Distance from Nucleolus (%)

**Supplementary figure 1.**

**Sodium azide treatment in M9 buffer results in similar VRK-1 and chromatin relocation as agarose pads and VRK-1 relocated to the NE in intestinal nuclei after sodium azide treatment**

**A.** Representative confocal single plane images of HIS-72::GFP and VRK-1::wrmScarlet after 30 minute treatment with 0.1% sodium azide in M9 buffer or levamisole, controls for Fig. 2D-F. **B.** Heatmaps displaying normalised intensity from nucleolus/nuclear centre to nuclear periphery for indicated immobiliser or fluorophore, where each row is an individual nucleus for images in A. (n>235 for all conditions). **C.** Average plot profiles for quantifications in B., measured as described in Fig. 1F. **D.** Single plane representative images of intestinal nuclei in L3/L4 larvae on either levamisole or sodium azide as described in A. Scale bar 20 µm. **E.** Heatmaps of normalized fluorescence intensity in intestinal nuclei, as described in B. n=42, n=41 for levamisole and n=27, n=24 for sodium azide 30 mins for HIS-72::GFP, VRK-1::wrmScarlet respectively. **F.** Average plot profiles of intestinal nuclei, as described in C.

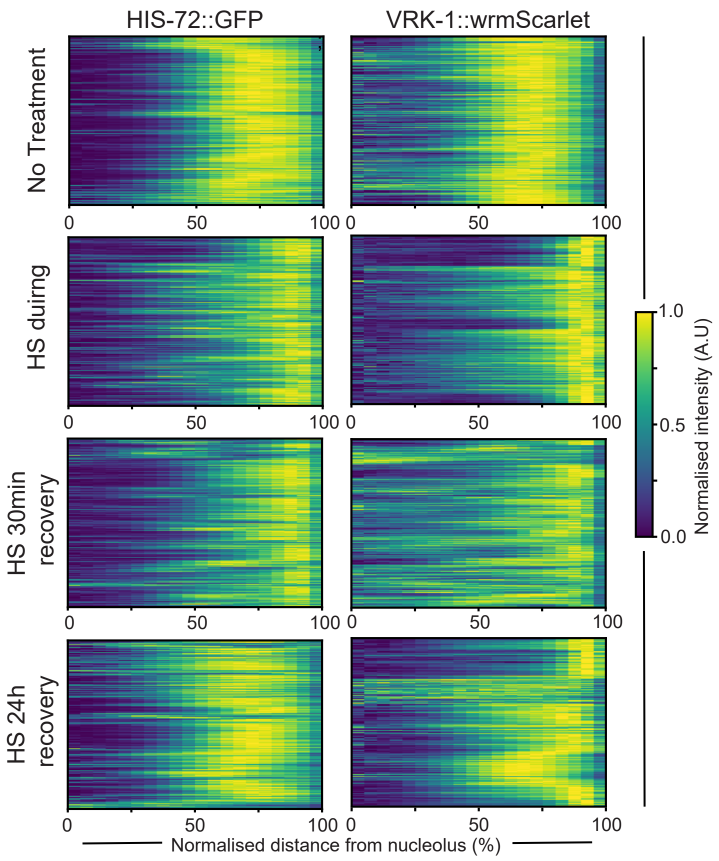
HIS-72::GFP
VRK-1::wrmScarlet
No Treatment
HS duirng
HS 30min recovery
HS 24h recovery
0
50
100
1.0
0.5
0.0
Normalised intensity (A.U)
Normalised distance from nucleolus (%)

**Supplementary figure 2.**
**Heatmaps for measurements described in Figure 3.**
Heatmaps displaying normalised signal intensity from nucleolus/nuclear centre to nuclear periphery for measurements of HIS-72::GFP and VRK-1::wrmScarlet at heat shock timepoints described shown in Fig.3BC. Each row indicates one nucleus.

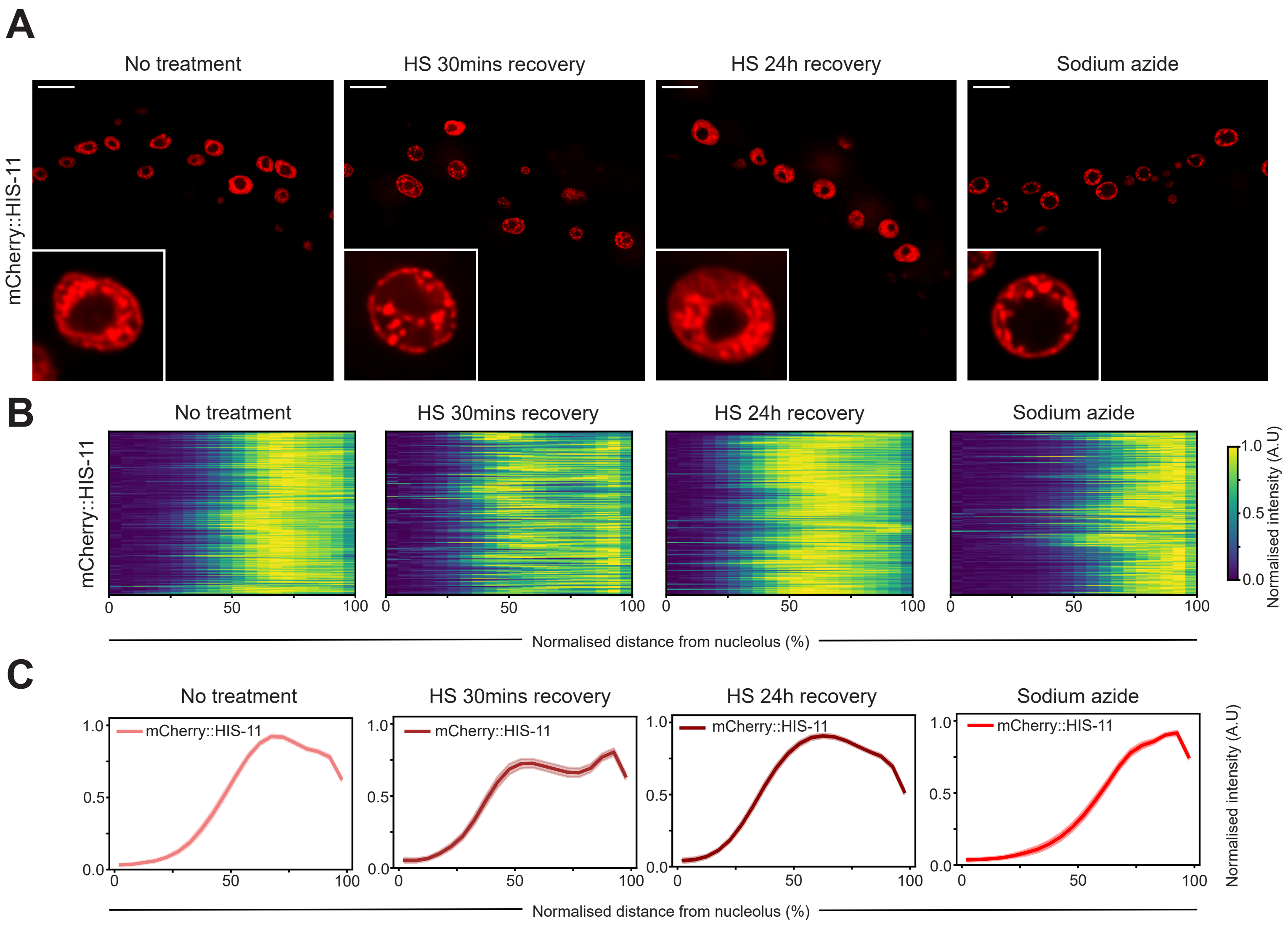

A
No treatment
HS 30mins recovery
HS 24h recovery
Sodium azide
mCherry::HIS-11
B
No treatment
HS 30mins recovery
HS 24h recovery
Sodium azide
mCherry::HIS-11
0
50
100
1.0
0.5
0.0
Normalised intensity (A.U)
Normalised distance from nucleolus (%)
C
No treatment
HS 30mins recovery
HS 24h recovery
Sodium azide
mCherry::HIS-11
1.0
0.5
0.0
0
50
100
Normalised intensity (A.U)
Normalised distance from nucleolus (%)

**Supplementary figure 3.**

**H2B (HIS-11) relocates in a similar manner to H3 in response to sodium azide treatment and heat stress.**

**A.** Representative single plane images of HIS-11::mCherry in hypodermal nuclei after treatment of levamisole, heat shock (30min recovery and 24h recovery) or sodium azide. Scale bar is equal to 10 µm. **B.** Heatmaps of normalised HIS-11::mCherry distribution from nucleolus/nucleus centre at conditions/timepoints stated in A., where each row corresponds to an individual nucleus. (n>235 for all conditions) **C.** Average plot profiles of HIS-11::mCherry as described in B.

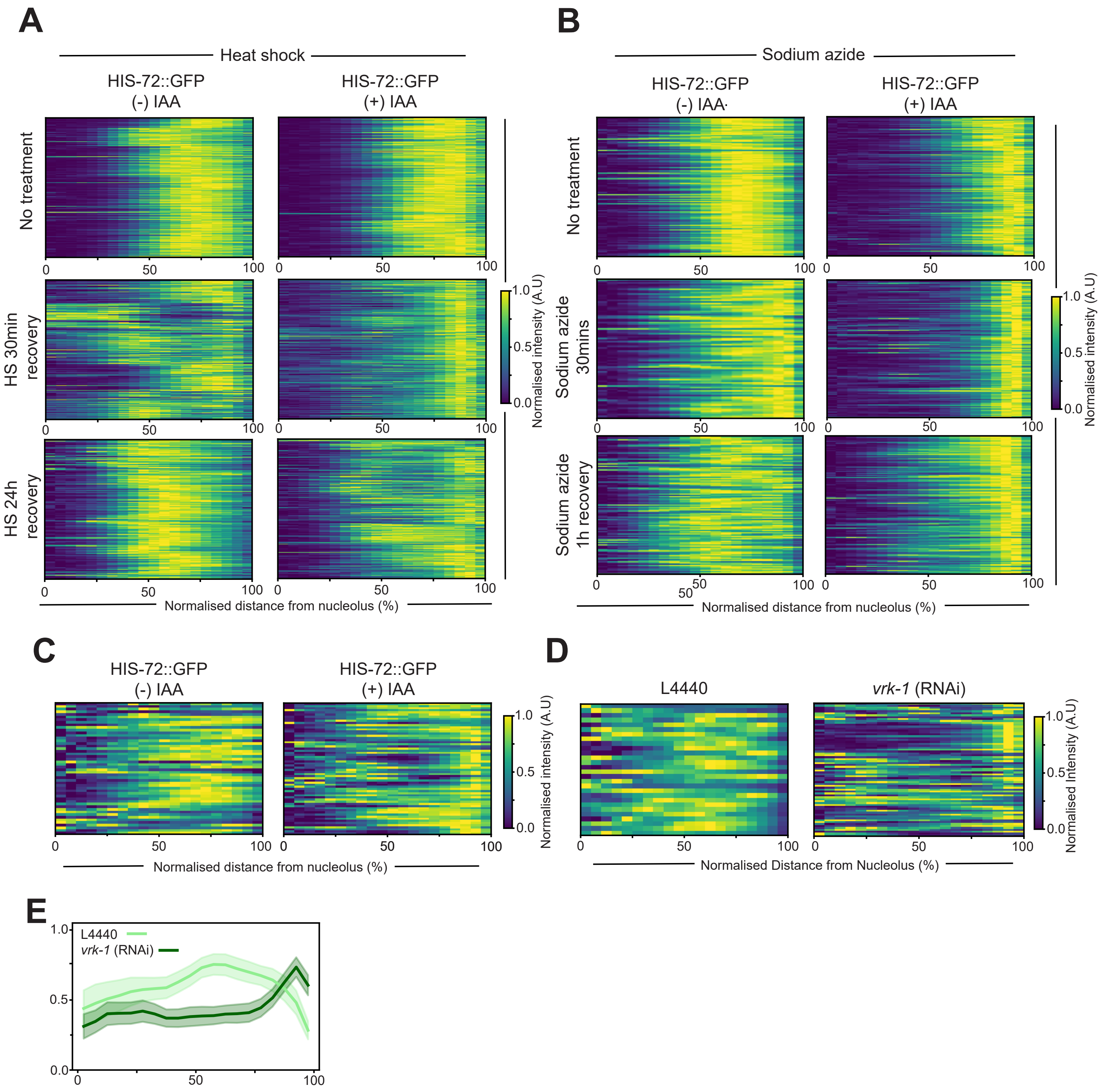
A
Heat shock
HIS-72::GFP (-) IAA
HIS-72::GFP (+) IAA
No treatment
HS 30min recovery
HS 24h recovery
Normalised intensity (A.U)
Normalised distance from nucleolus (%)
B
Sodium azide
HIS-72::GFP (-) IAA
HIS-72::GFP (+) IAA
No treatment
Sodium azide 30mins
Sodium azide 1h recovery
Normalised intensity (A.U)
Normalised distance from nucleolus (%)
C
HIS-72::GFP (-) IAA
HIS-72::GFP (+) IAA
Normalised intensity (A.U)
Normalised distance from nucleolus (%)
D
L4440
vrk-1 (RNAi)
Normalised Intensity (A.U)
Normalised Distance from Nucleolus (%)
E
L4440
vrk-1 (RNAi)

**Supplementary figure 4.**
**Heatmaps related to figure 4**
**A.** Heatmaps displaying quantifications of normalised signal intensity of HIS-72::GFP from nucleolus/nuclear centre to nuclear periphery for Fig.4BC at indicated timepoints before and after heat shock. Nuclear distribution measured as described in 1E. **B.** Heatmaps displaying quantifications of normalised signal intensity of HIS-72::GFP from nucleolus/nuclear centre to nuclear periphery for Fig.4EF at indicated timepoints before and after sodium azide treatment in M9 buffer. **C.** Heatmaps showing quantifications of individual nuclei for day 7 adults, related to Fig. 4GH. **D.** Heatmaps showing quantifications of individual nuclei for day 7 adults after depletion of VRK-1 by RNAi. **E.** Average plot profiles related to D.

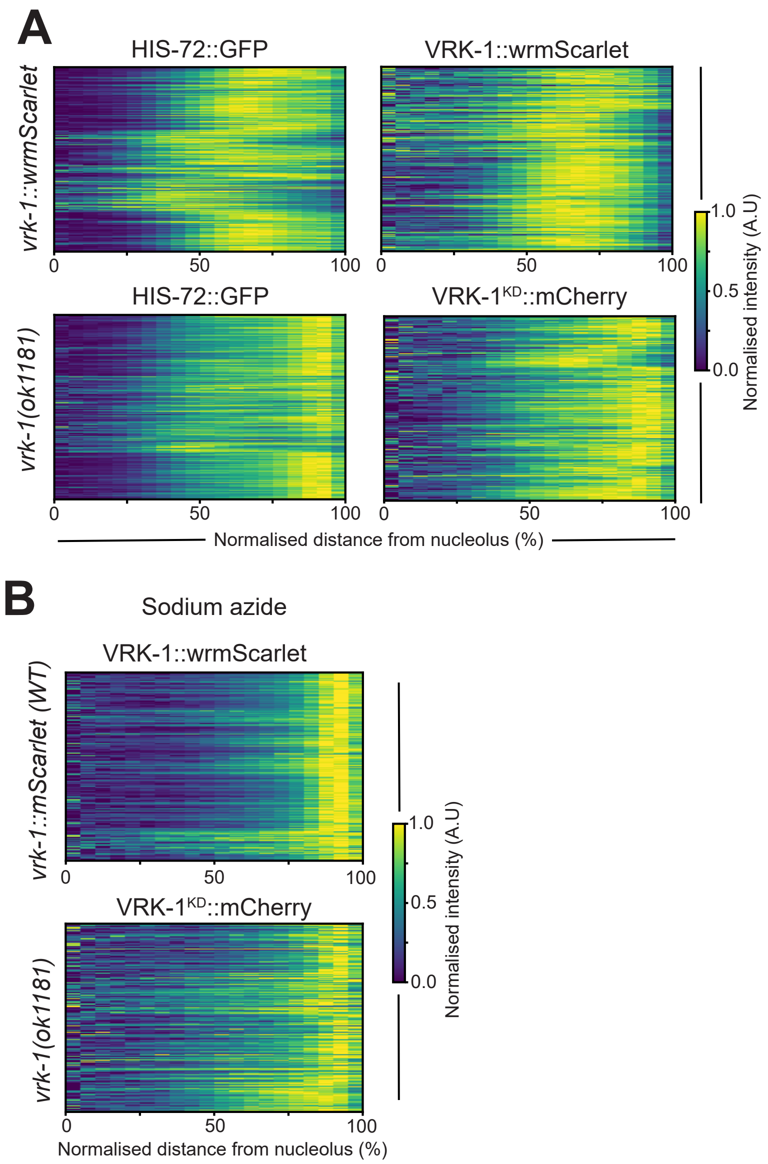
A
HIS-72::GFP
VRK-1::wrmScarlet
vrk-1::wrmScarlet
HIS-72::GFP
VRK-1KD::mCherry
vrk-1(ok1181)
0
50
100
1.0
0.5
0.0
Normalised intensity (A.U)
Normalised distance from nucleolus (%)
B
Sodium azide
VRK-1::wrmScarlet
vrk-1::mScarlet (WT)
VRK-1KD::mCherry
vrk-1(ok1181)
Normalised intensity (A.U)
Normalised distance from nucleolus (%)

**Supplementary figure 5.**
**Heatmaps for quantifications from Figure 5.**
**A.** heatmaps displaying quantifications of HIS-72::GFP/VRK-1::wrmScarlet (n=352, n=256, respectively) (top) and HIS-72::GFP/VRK-1KD::mCherry in *vrk-1(ok1181)* background (n=459, n=222, respectively) (bottom) of nuclear distribution as described in 1E. **B.** Heatmaps of radial fluorescent distribution of nuclei described in A., where each row represents an individual nuclei. n=219, n=230 for VRK-1::wrmScarlet and VRK-1KD::mCherry, respectively).

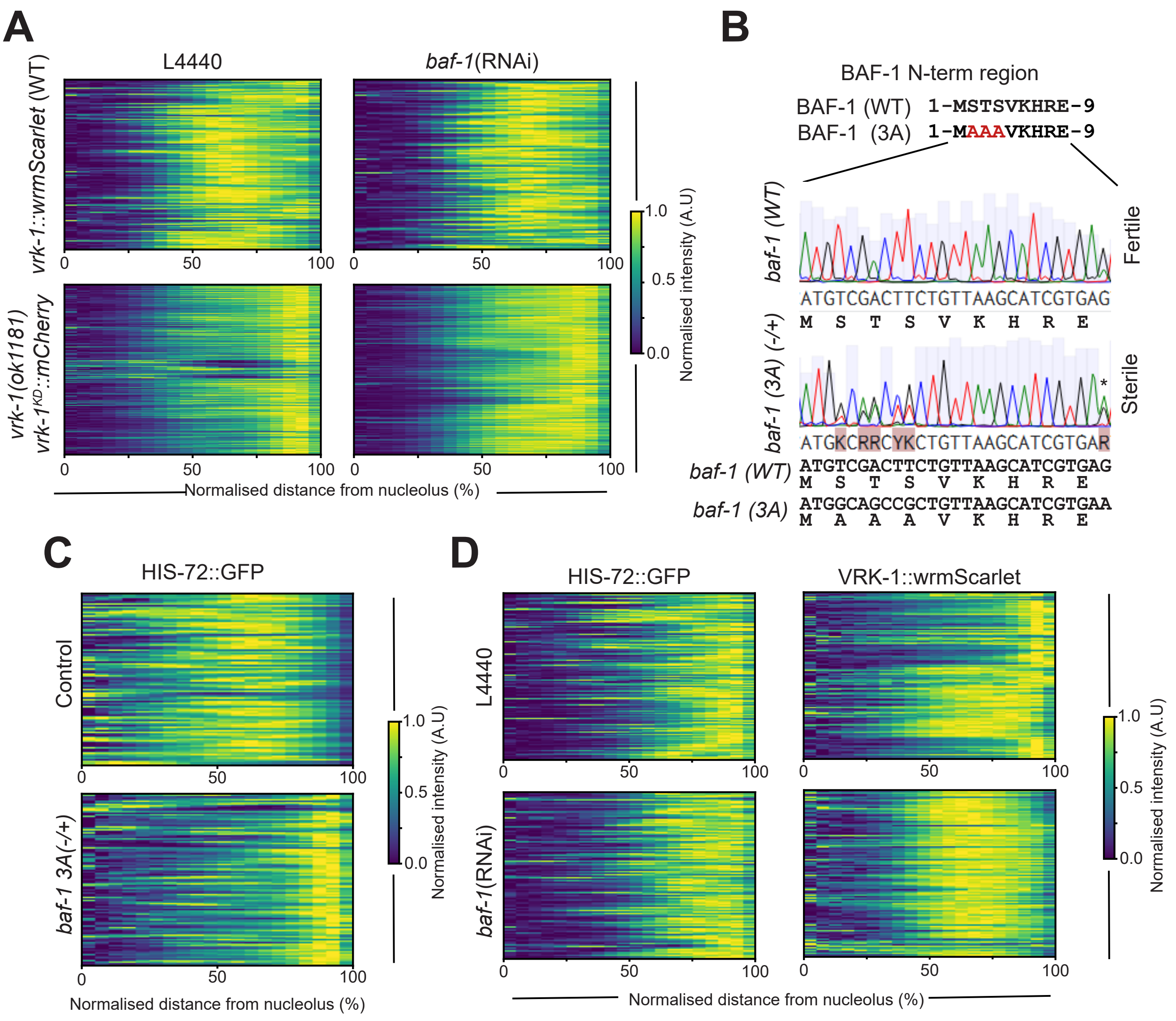
A
L4440
baf-1(RNAi)
vrk-1::wrmScarlet (WT)
vrk-1(ok1181) vrk-1^KD::mCherry
Normalised intensity (A.U)
Normalised distance from nucleolus (%)
B
BAF-1 N-term region
BAF-1 (WT) 1-MSTSVKHRE-9
BAF-1 (3A) 1-MAAAVKHRE-9
baf-1 (WT)
Fertile
ATGTCGACTTCTGTTAAGCATCGTGAG
M S T S V K H R E
baf-1 (3A) (-/+)
Sterile
ATGKCRRCYKCTGTTAAGCATCGTGAR
baf-1 (WT) ATGTCGACTTCTGTTAAGCATCGTGAG
M S T S V K H R E
baf-1 (3A) ATGGCAGCCGCTGTTAAGCATCGTGAA
M A A A V K H R E
C
HIS-72::GFP
Control
baf-1 3A(-/+)
Normalised intensity (A.U)
Normalised distance from nucleolus (%)
D
HIS-72::GFP
VRK-1::wrmScarlet
L4440
baf-1(RNAi)
Normalised intensity (A.U)
Normalised distance from nucleolus (%)

**Supplementary figure 6.**

**Heatmaps for quantifications related to Figure 6.**
**A.** Heatmaps showing normalised signal intensity of HIS-72::GFP, where each row indicates an individual nucleus, related to Fig. 6AB. **B.** Schematic showing the substituted amino acids in BAF-1 3A mutant. Sanger sequencing results showing that F1 worms are heterozygote for BAF-1 3A mutation. Note: heterozygotes animals are sterile. Starred mutation at 3’ region is silent, and part of the mutation to ablate the PAM site. **C.** Heatmaps showing normalised signal intensity of HIS-72::GFP, where each row indicates an individual nucleus in WT BAF-1 nuclei (control) and *baf-1* 3A heterozygotes, related to quantification in Fig. 6DE. Genotype was inferred from sterility phenotype, as described in the methods section. **D.** Heatmaps showing normalised signal intensity of HIS-72::GFP, where each row indicates an individual nucleus related to quantifications from Fig. 6FG.
